# Neutrophils in pancreatic cancer: Potential therapeutic targets

**DOI:** 10.3389/fonc.2022.1025805

**Published:** 2022-10-17

**Authors:** Wenkai Jiang, Xin Li, Caifei Xiang, Wence Zhou

**Affiliations:** ^1^ The First Clinical Medical College, Lanzhou University, Lanzhou, China; ^2^ The Second Clinical Medical College, Lanzhou University, Lanzhou, China; ^3^ Department of General Surgery, The Second Hospital of Lanzhou University, Lanzhou, China

**Keywords:** anticancer therapy, neutrophil, neutrophil extracellular trap, pancreatic cancer, tumor-associated neutrophil

## Abstract

Pancreatic cancer is a digestive system malignancy and poses a high mortality worldwide. Traditionally, neutrophils have been thought to play a role in acute inflammation. In contrast, their importance during tumor diseases has been less well studied. Generally, neutrophils are recruited into the tumor microenvironment and exert inflammation and tumor-promoting effects. As an essential part of the tumor microenvironment, neutrophils play diverse roles in pancreatic cancer, such as angiogenesis, progression, metastasis and immunosuppression. Additionally, neutrophils can be a new potential therapeutic target in cancer. Inhibitors of cytokines, chemokines and neutrophil extracellular traps can exert antitumor effects. In this review, we describe the role of neutrophils in the development and progression of pancreatic cancer, discuss their potential as therapeutic targets, and aim to provide ideas for improving the prognosis of patients with this malignant tumor disease.

## Introduction

**Figure 1:**
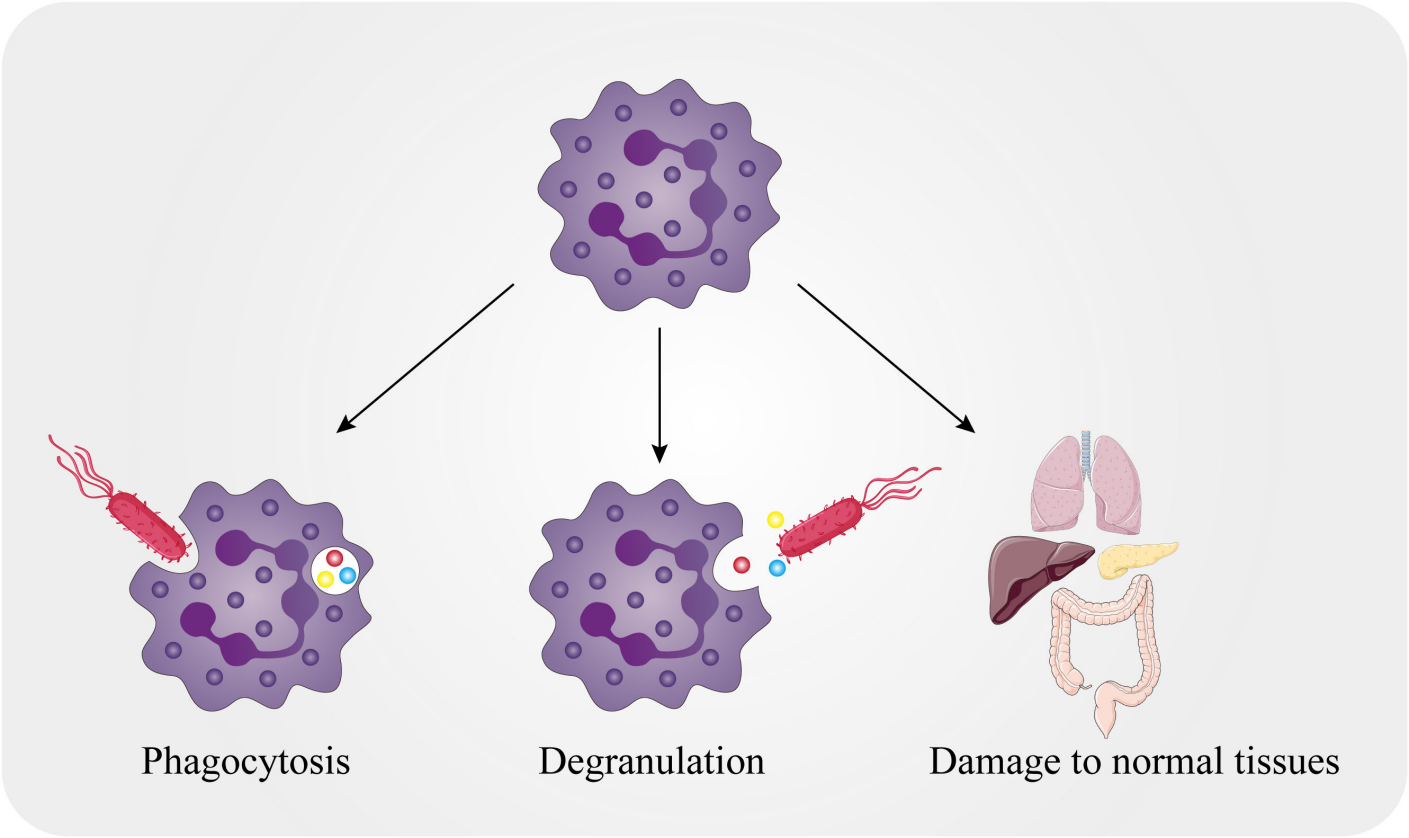
Killing mechanisms of neutrophils. The killing mechanisms of neutrophils include both intracellular and extracellular mechanisms. Neutrophils can encase pathogens in the phagocytic body through phagocytosis and can also release particles into the extracellular environment that act on external pathogens. Meanwhile, normal tissues in the body can be attacked by neutrophils.

The tumor microenvironment (TME) is a vital part of tumor formation, and TME homeostasis is regulated by signal transduction pathways and metabolism among tumor cells, endothelial cells, stromal cells and immune cells (1). Tumor growth can be modulated by the secretion of signaling molecules by immune cells, so in some cases tumor growth, invasion and metastasis can be regulated by the interactions between cancer cells and immune cells in the TME (2). Therefore, it is important to understand the relationships between cancer cells and the TME for the development of effective therapies. Traditionally, neutrophils have been thought to play a role in acute inflammation. Increased numbers of neutrophils enter tissues and kill microorganisms by phagocytosis or the release of active substances from granules. Moreover, these cells can also cause severe damage to normal tissues (**Figure 1**). With increased knowledge of neutrophils, it has been found that neutrophils also participate in chronic inflammation, adaptive immune responses and tumor diseases (3, 4). Tumor-associated macrophages and fibroblast cells are involved in inflammation, which can support cancer progression (5). However, some evidence also suggests that neutrophils can be a new example of cancer-related inflammation and immunity (6, 7).

Pancreatic cancer is a malignancy and poses a serious medical trouble. In 2017, the number of pancreatic cancer cases worldwide was more than twice as high as in 1990. There was a 2.3-fold increase in the number of deaths from 196,000 in 1990 to 441,000 in 2017 (8). Despite progress in the available treatment methods and efficacy, pancreatic cancer patients have poor prognosis. Studies on the carcinogenic mechanism and the search for immune targets based on the TME have become directions in pancreatic cancer research, and tumor-associated neutrophils (TANs) provide new ideas.

In this review, we summarize the functions of neutrophils in pancreatic cancer development processes, such as angiogenesis, progression, metastasis, and immunosuppression. Next, we discuss the potential of neutrophils as anticancer therapeutic targets. We also propose future directions and how neutrophils may affect the therapeutic outcomes of pancreatic cancer patients, which may contribute to a new generation of anticancer therapies for pancreatic cancer patients.

## Neutrophil life activity

### Production, differentiation and death

Neutrophils account for 50-70% of circulating leukocytes in the human body (9). More than 10^11^ neutrophils may be produced each day (3). Human neutrophils have a half-life of approximately 8 hours in the circulation and are generally considered short-lived cells, but some studies have shown that the average circulating life of human neutrophils is 5.4 days (3, 10, 11). As hematopoietic stem cells differentiate in bone marrow, they first give rise to common myeloid progenitors, followed by granulocytes and monocytes. Granulocyte-colony stimulating factor (G-CSF), produced by bone marrow stromal cells, is a key cytokine that stimulates the production and mobilization of neutrophils in bone marrow (12). G-CSF regulates the differentiation of granulocyte-monocyte progenitor cells into neutrophils and the formation of myeloblasts. The subsequent stages of differentiation are promyelocytic, myelocyte, metamyelocyte, band cell and polymorphonuclear granulocyte (3). Mature neutrophils are released into the bloodstream and play roles in inflammation, infection, and chronic diseases in the body.

There are protective mechanisms to balance the number of neutrophils so that these cells do not become overactive in blood vessels and cause severe damage to normal tissues. The bone marrow is also a site at which circulating neutrophils are recycled. Neutrophil release is negatively regulated by CXC-chemokine receptor 4 (CXCR4) signaling in a cellular autonomous manner (13). In the bone marrow, senescent neutrophils are removed *via* a CXCR4-dependent process. Neutrophils are reassigned from the bone marrow to the blood when CXCR4 signaling is lost (14). In addition, liver Kupffer phagocytosis functions and regulation of the microbiome also limit over the numbers of neutrophils (15, 16). Neutrophils are often activated by a two-step process of priming followed by activation, which avoids non-specific triggering of their cytotoxic mechanisms, and undergo rapid apoptosis which blocks their ability to respond to extracellular ligands (17, 18). Thus, these mechanisms by which both the number and activation of neutrophils are tightly controlled in the circulation ensures that the human body is protected against microbial pathogens and reduces damage to its own tissues.

### Neutrophil recruitment

Neutrophil recruitment begins with changes in endothelial cells and processing including tethering, rolling, adhesion, crawling and, finally, transmigration (4). After neutrophils reach the vascular edge, their rolling process is dependent on selectin, and the adhesion process is dependent on integrin, which results in the tight adherence of neutrophils to endothelial cells (19, 20). Platelet endothelial cell adhesion molecule is located on the neutrophil surface and endothelial cell surface, promoting neutrophil migration out of the vascular endothelium by mediating the binding of these two cells (21). Then neutrophils secrete molecules such as collagenase to degrade the vascular basement membrane but preferentially they move through membrane regions with low expression of extracellular matrix components, and then enter the surrounding tissues (4). Then, neutrophils migrate between pericytes, crawl along the cells through intercellular signals and search for gaps through which they can finally leave the vasculature. After extravasation, neutrophils make directional movements along the chemical concentration gradient and accumulate in inflammatory sites.

Chemokines, such as CXCR1, CXCR2 and CXCR4, are critical in neutrophil recruitment (4, 22, 23). In addition, cytokines such as interleukins (ILs) and tumor necrosis factors (TNFs), and intracellular proteins such as poly ADP-ribose polymerase 1, cathepsin C and S100 calcium-binding protein A9 can also increase neutrophil activation and recruitment (24–27). Prolongation of the neutrophil lifespan can further enhance their functional roles. Although the normal lifespan of neutrophils is short, certain cytokines and bacterial products can prolong neutrophil survival by interfering with apoptosis. For instance, G-CSF can delay neutrophil apoptosis by inhibiting the activation of calpain, a calcium-dependent cysteine protease that is upstream of caspase-3, resulting in a delay in apoptosis of approximately 12 hours (28, 29).

## Neutrophils and cancer

### Circulating neutrophils

Serological indicators are widely used to predict the overall survival (OS, a term that denotes the time of staying alive for individuals that suffer from a specific disease) of tumor diseases due to their advantages of simplicity, economy and noninvasiveness (30). As a marker of systemic inflammation, the neutrophil/lymphocyte ratio (NLR) is currently an attractive biomarker for risk stratification and guiding treatment decisions in cancer patients (31).

Especially in early-stage pancreatic ductal adenocarcinoma (PDAC), finding biomarkers to predict recurrence can lead to a better prognosis. The NLR is associated with the tumor stage, and patients with PDAC whose tumor stages were less than IIA had longer OS and recurrence-free survival (RFS, time from surgery to the date of first recurrence) when the NLR > 2.2 (hazard ratio =3.310, 95% confidence interval: 1.259-8.745). However, the NLR was not associated with OS or RFS in patients with tumor stage greater than IIB (32). Since only a few pancreatic cancer patients have surgical indications at the time of diagnosis, it is important to construct accurate prognostic models in patients with unresectable pancreatic cancer. The NLR is better than other serological indicators (such as the platelet/lymphocyte ratio and prognostic nutritional index) among prognostic factors in nonsurgically resectable pancreatic cancer patients after 6 months of follow-up. Multivariate analysis showed that a high NLR (HR=2.430, 95% CI: 1.484 to 3.977) is an independent predictor of OS (33).

The NLR can also predict how pancreatic cancer will respond to drug treatment. In retrospective studies of pancreatic cancer patients treated with FOLFIRINOX (oxaliplatin, irinotecan, leucovorin, 5-fluorouracil) and immune checkpoint inhibitors, a high NLR was associated with poor prognosis (34, 35).

### Neutrophil extracellular traps (NETs)

NETs are formed by the release of cellular contents by activated neutrophils into surrounding tissues or circulation (36). Neutrophils can be stimulated to produce NETs by a variety of substances, including bacteria, viruses and some chemical or biological factors (37). Chemokines in the TME, such as IL-8, can promote the formation of NETs and help recruit more neutrophils (38). Conversely, NETs can also promote tumor growth and have a positive effect on the TME, including enhancing mitochondrial function in tumor cells, blocking the function of immune cells and exhibiting angiogenic activity by increasing capillary length, loop number, and tubule area (39, 40). Thus, NETs can accelerate tumor growth and cause tumor immune escape.

Based on the putative role of NETs in the TME, NETs may have potential as a biomarker for prognosis of some cancer patients. For example, the level of NETs is increased in cancer patients and is significantly higher in patients with advanced stage disease than in patients with early disease (41). In patients with pancreatic cancer, NET was an independent prognostic factor for OS (HR=2.366, 95% CI: 1.408–3.978) and RFS (HR=3.037, 95% CI: 1.809–5.098) and could predict the survival of patients who received gemcitabine-based chemotherapy (42). At present, NETs were confirmed to be involved in the biological process of pancreatic cancer in some preclinical studies. NETs-mediated metastasis and drug resistance of cancer cells will provide new insights into anticancer therapies.

### TANs and cancer diseases

TAN can be involved in the progression of tumor disease. A meta-analysis showed that the level of intratumoral neutrophils was independently associated with OS and RFS in cancer patients (43). In the TME, TANs can take an antitumorigenic and pro-tumorigenic phenotype (44). Cytokines in the TME impacts on the balance of these two subpopulations. For instance, TAN can become the “promoting the tumor” type in response to transforming growth factor-β (TGF-β) (45). Neutrophil with this phenotype can produce pro-tumor factors (6). By contrast, low doses of interferon -β induced neutrophil to polarized to the “antitumor” phenotype in C57BL/6 and BALB/C mice, and similar changes were also observed in melanoma patients treated with type I interferon (46). “Antitumor” phenotype TANs produce chemokines, such as CCL3, CXCL9, CXCL10, to recruit CD8+ T cells to the TME (47). There is also evidence that they can increased cytotoxicity and reduced immunosuppression by the production of TNF-α, ROS and CD95, thus providing anticancer effect (48, 49).

The diversity of neutrophils leads to their dual potential in the TME. As a part of tumor-associated inflammation, TANs are involved in tumor growth and metastasis. Additionally, neutrophils can interact with other immune cells and stromal cells, resulting in extracellular matrix accumulation and immune function changes (50). In mouse models, TANs mediate the infiltration of regulatory T (Treg) cells and macrophages in the TME by secreting the chemokines CCL2 and CCL17, leading to the growth of hepatocellular carcinoma cells and increasing the resistance of hepatocellular carcinoma patients to sorafenib (51). In colorectal cancer, tumor growth is related to the gut microbiome because these tumor cells produce IL-17 and mediate the inflammatory response (e.g., driving B-cell infiltration). Neutrophils can limit the number of microorganisms and the expression of IL-17 to reduce inflammation related to tumor progression (52). Conversely, neutrophils can also mediate antitumor responses (50). IL-1 and IL-1β signaling in neutrophils enhances the antimicrobial activities in colorectal cancer, which inhibits bacterial-driven inflammation and alleviated tumorigenesis (53). Hepatocyte growth factor (HGF) acts on the HGF receptor expressed on neutrophils and promotes the production of inducible nitric oxide synthase (iNOS); iNOS releases nitric oxide and promotes apoptosis of tumor cells (54).

## Carcinogenic mechanisms of neutrophils in pancreatic cancer

As we mentioned earlier, TANs participate in various cancer-related processes in the TME and are associated with poor prognosis for most cancers. In this section, we will introduce how TANs promote the progression of pancreatic cancer, such as through angiogenesis, pancreatic cancer cell metastasis and immune suppression. A deep understanding of these mechanisms will not only enable us to understand the promoting effect of the TME on pancreatic cancer, but also provide us with new therapeutic targets.

### TANs and angiogenesis

Angiogenesis is a critical link in tumor growth and metastasis, and is jointly regulated by tumor cells, stromal cells and their bioactive products, such as various growth factors and extracellular matrix (55). Activated neutrophils release multiple angiogenic factors, including vascular endothelial growth factors (VEGFs), CXCLs and matrix metalloproteinases (MMPs), and form NETs (56).

Histones, which are major components of NETs, significantly increased vascular endothelial tubule formation in a dose-dependent manner (57). After treatment with 100 IU/mL heparin and 62.5 μg/mL polysialic acid for 1 hour, the histone-induced production of tubules in vascular endothelial cells was inhibited. This effect occurred because heparin and polysialic acid are anionic substances that bind to positively charged histones and neutralize their activity (57).

MMP plays an important role in angiogenesis, and MMP-9 promotes the release of VEGF from the extracellular matrix and participates in the interaction between VEGF and VEGF receptors (58). Neutrophils can be a source of MMP-9 in tumor angiogenesis (59). The addition of neutrophils to pancreatic cancer cells can increase the budding rate by more than 2.5 times because MMP-9 may promote endothelial cell migration. After 14 days of treatment with bevacizumab (a VEGF inhibitor) and doxycycline (a drug which could inhibit angiogenesis as effectively as MMP-9 inhibitors), the tumor volume in pancreatic cancer mice was significantly reduced. Furthermore, the average vascular density of pancreatic cancer mice was also significantly reduced (60). Therefore, MMP-9 produced by neutrophils may be a therapeutic target in pancreatic cancer treatment and provide a feasible alternative treatment for pancreatic cancer patients.

Neutrophil gelatinase-associated lipocalin (NGAL) is secreted by neutrophils and is upregulated in a variety of tumor diseases (61). NGAL can potentially inhibit angiogenesis by reducing VEGF production in pancreatic cancer cells. Compared with that in the control groups, adding NGAL reduced the tube formation of human umbilical vein endothelial cells (HUVECs) in MIA PaCa-2 (RRID: CVCL_0428) and PANC-1 (RRID: CVCL_0480) (two pancreatic cancer cell lines) cells by 69.5% ± 5% and 68% ± 7.5%, respectively (62). Moreover, CXCL5 mediates pancreatic cancer angiogenesis in mouse model by activating multiple signaling pathways, including signal transducer and activator of transcription pathways and extracellular signal–regulated kinase pathways in human endothelial cells (63). Due to the important role of neutrophils in tumor angiogenesis, neutrophil suppression may be an effective anticancer strategy.

### Progression and metastasis

TANs are involved in tumor progression and migration. As early as 1989, neutrophils were shown to promote lung metastasis of breast cancer (64). TNF, leukotriene B4 and IL in the TME play roles in tumor progression and metastasis influenced by neutrophils (6).

In normal pancreas tissue, obesity promotes the inflammatory response and fibrosis; in pancreatic cancer, cytokines produced by dysfunctional fat cells, such as IL-1β, increase pancreatic stellate cells (PSC) activation and recruit TANs (65). PSCs are the main cell type in the pancreatic cancer stroma, and their large presence suggests that they may contribute to the metabolism of cancer cells (66). TANs also secrete IL-1β, which is involved in PSC activation, immunosuppression and PDAC progression. Moreover, adjuvant chemotherapy showed no significant survival advantage in overweight and obese patients with PDAC; Thus, the cross interaction between adipocytes, TANs and PSCs promotes the progression of PDAC, with IL-1β playing a major role in this process (65).

The purinergic receptor P2RX1, an ATP-gated ion channel, is associated with the inflammatory activation of immune cells (67). A large number of P2RX1-deleted neutrophils were found in the hepatic metastasis model of PDAC. The immune response of P2RX1 negative neutrophils in the PDAC TME is characterized by elevated MMP-9. The metabolic characteristics were a significant increase in the oxygen consumption rate and a nonsignificant increase in the extracellular acid rate (decreased glycolysis in neutrophils and enhanced oxidative phosphorylation in mitochondria). This effect occurs because the deletion of P2RX1 can increase the activity of the neutrophil transcription factor NF-E2 p45-related factor 2 (NRF2) (68). NRF2 is critical in regulating redox, metabolic, protein homeostasis, and inflammation (69). Increased NRF2 activity contributes to the metabolic reprogramming of neutrophils during polarization. Second, NRF2 directly regulates PD-L1 transcription and has a direct impact on CD8+ T-cell failure (68). Because Nrf2 is critical for immunosuppressive microenvironment formation in pancreatic cancer liver metastases *via* shaping the immunosuppressive phenotypes of P2RX1-negative neutrophil, future therapy, such as inhibiting the specific gene to reduce the particular phenotype of neutrophil subpopulation, may help treat pancreatic cancer.

**Figure 2:**
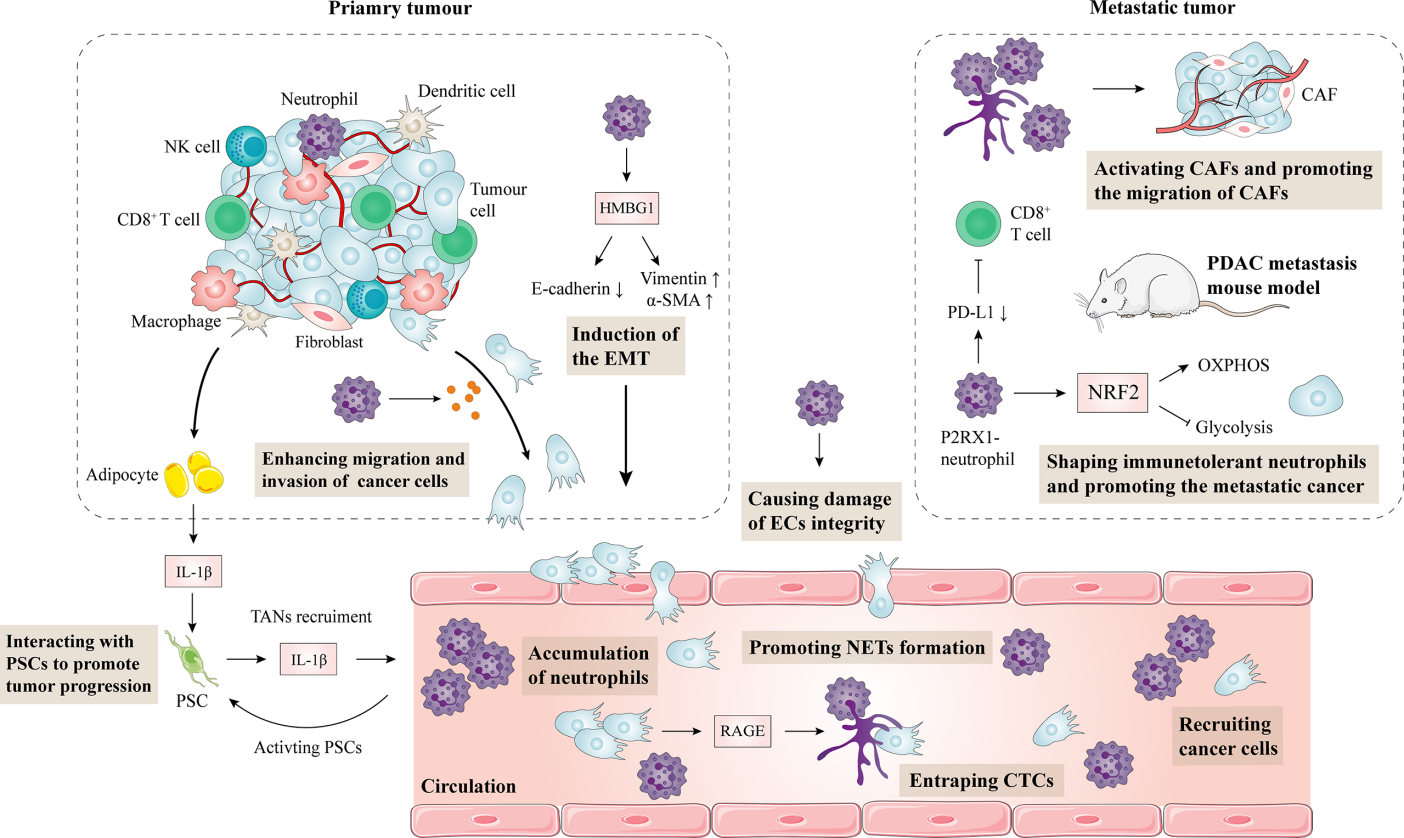
Impact of neutrophils on the progression and metastasis of pancreatic cancer. Obesity-induced inflammation and TAN infiltration activate PSCs, leading to connective tissue proliferation in the TME and promoting tumor growth. Conversely, PSCs can also recruit TANs. Neutrophils produce HMGB1 in pancreatic cancer, which induces the epithelial-mesenchymal transformation of pancreatic cancer. Moreover, neutrophils can also secrete elastase to degrade E-cadherin on pancreatic cancer cells, resulting in the enhanced migration and invasion of pancreatic cancer cells. In a mouse metastatic tumor model, NRF2 activity in P2RX1 negative neutrophils is elevated, leading to metabolic reprogramming during polarization. As a result, CD8+ T cells are inhibited, and tumor immune escape is mediated. NETs are upregulated in pancreatic cancer through a RAGE dependent and autophagy mediated pathway. NETs enhance the migration of hepatic stellate cells, activate cancer-associated fibroblasts, and promote hepatic metastasis of pancreatic cancer. Neutrophils are also involved in pancreatic cancer vascular endothelial cell integrity damage and promote metastasis of pancreatic cancer cells. CAF, cancer-associated fibroblasts; EMT, epithelial-mesenchymal transformation; PDAC, pancreatic ductal adenocarcinoma; PD-L1, programmed cell death-ligand 1; PSC, pancreatic stellate cell; IL, interleukin; EC, endothelial cell; NET, neutrophil extracellular trap.

NET formation is dependent on receptor for advanced glycation end products (RAGE) and autophagy pathways and is mediated by citrullination of histones to allow DNA expulsion from cells. Inhibition of autophagy by chloroquine or ablation of RAGE resulted in decreased NET formation (70). NETs can enhance tumor migration and invasion by inducing epithelial cells to transform into mesenchymal cells. Moreover, neutrophils can degrade E-cadherin on pancreatic cancer cells by secreting elastase, leading to increased tumor cell migration and invasion, and resulting in PDAC progression and metastasis (71). The effects of neutrophils on the progression and metastasis of pancreatic cancer are summarized in **Figure 2**.

### TANs and immunosuppression

The TME has the ability to regulate immunosuppression, and understanding the mechanisms by which pancreatic cancer cells evade tumor immunity is crucial for developing more effective therapies. NETs can promote tumor growth and metastasis through a variety of mechanisms: trapping circulating tumor cells and protecting them, thereby preventing T cell and natural killer cell-mediated cytotoxicity (56).

CXCL5, a CXCR2 ligand, are significantly elevated in pancreatic cancer and can recruit TANs. This process is regulated by the activity of the NF-κB signaling pathway in mouse models, suggesting that neutrophils are involved in pancreatic cancer inflammation. Reducing CXCR2 significantly inhibited the number of TANs in pancreatic cancer, leading to spontaneous, T-cell-dependent tumor growth inhibition (72). Because the CXCR2 ligand axis is involved in the recruitment of TANs and the regulation of T-cell immunity in pancreatic cancer, it is expected to be a potential therapeutic target for pancreatic cancer.

Programmed cell death protein 1 (PD-1) and cytotoxic T-lymphocyte-associated protein 4 (CTLA-4) are both signaling molecules commonly seen on activated T cells, and have been found to be effective immunotherapeutic targets in cancer (73). IL-17 is highly expressed in tumor tissues. After treatment with IL-17, several chemokines capable of recruiting neutrophils were significantly induced. Researchers found that IL-17 signaling favors CD8+ T-cell inactivation and significantly affects immune checkpoint blockade (PD-1, CTLA-4) sensitivity. However, inhibition of neutrophils neutralizes IL-17. Thus, IL-17 promotes immunosuppression and resistance to immune checkpoint blockade by inducing neutrophil infiltration in pancreatic cancer (74).

Heterogeneous myeloid-derived suppressor cells (MDSCs) are suppressors of antitumor immunity, making tumor immunotherapy difficult. MDSC can inhibit T cell and NK cell proliferation and promote the function of Treg cells by secreting TGF-β and IL-10; moreover, MDSC can release reactive oxygen species and cause damage to the infiltrating lymphocytes in the TME (75). Neutrophil-like MDSCs (nMDSCs) are significantly increased in PDAC. The expression of high levels of CD13 on nMDSCs more effectively suppresses antitumor immunity through an arginase-1-related mechanism and PDAC patients with higher CD13 expression have a shorter OS (76).

Currently, there have been few studies on the specific mechanism by which TAN indirectly promotes immunosuppression. TANs can promote the recruitment of Tregs to the TME through the release of the chemokine CCL17, leading to the formation of an immunosuppressive microenvironment (77). A link between TANs and other immunosuppressive cell types acting together to impair antitumor immunity in pancreatic cancer needs to be further studied in the future. The interactions between neutrophils and other cells in the pancreatic cancer TME are summarized in **Table 1**.

**Table 1:** Interaction of neutrophils with other cells in the pancreatic cancer TME.

Factors	Source	Responder	Effects	Ref.
MMP-9	Neutrophil	EC	Promoting EC migration	(60)
NGAL	Neutrophil	EC	Reducing VEGF secretion	(62)
NRF2	Neutrophil	CD8+ T cell	Regulating immune checkpoint transcription	(68)
CXCL5	Most cells	EC	Mediating angiogenesis in pancreatic cancer	(63)
IL-17	CD4+ T cell	• Neutrophil • T cell	Suppression of antitumor immunity	(74)
IL-1β	• Neutrophil • PSC	• PSC • Neutrophil	Promoting inflammation in PDAC and enhancing chemotherapeutic resistance	(65)
TGF-β	Most cells	Neutrophil	Promoting neutrophils to become the “pro-tumor” phenotype	(45)

TME, tumor microenvironment; Ref., reference; MMP-9, matrix metalloproteinase-9; EC, endothelial cells; NGAL, neutrophil gelatinase-associated lipocalin; VEGF, vascular endothelial growth factor; NRF2, neutrophil transcription factor NF-E2 p45-related factor 2; PD-L1, programmed cell death-ligand 1; CXCL, CXC-chemokine ligand; IL, interleukin; PSC, pancreatic stellate cell; PDAC, pancreatic ductal adenocarcinoma; TGF-β, transforming growth factor-β.

## Neutrophils as therapeutic targets

**Figure 3:**
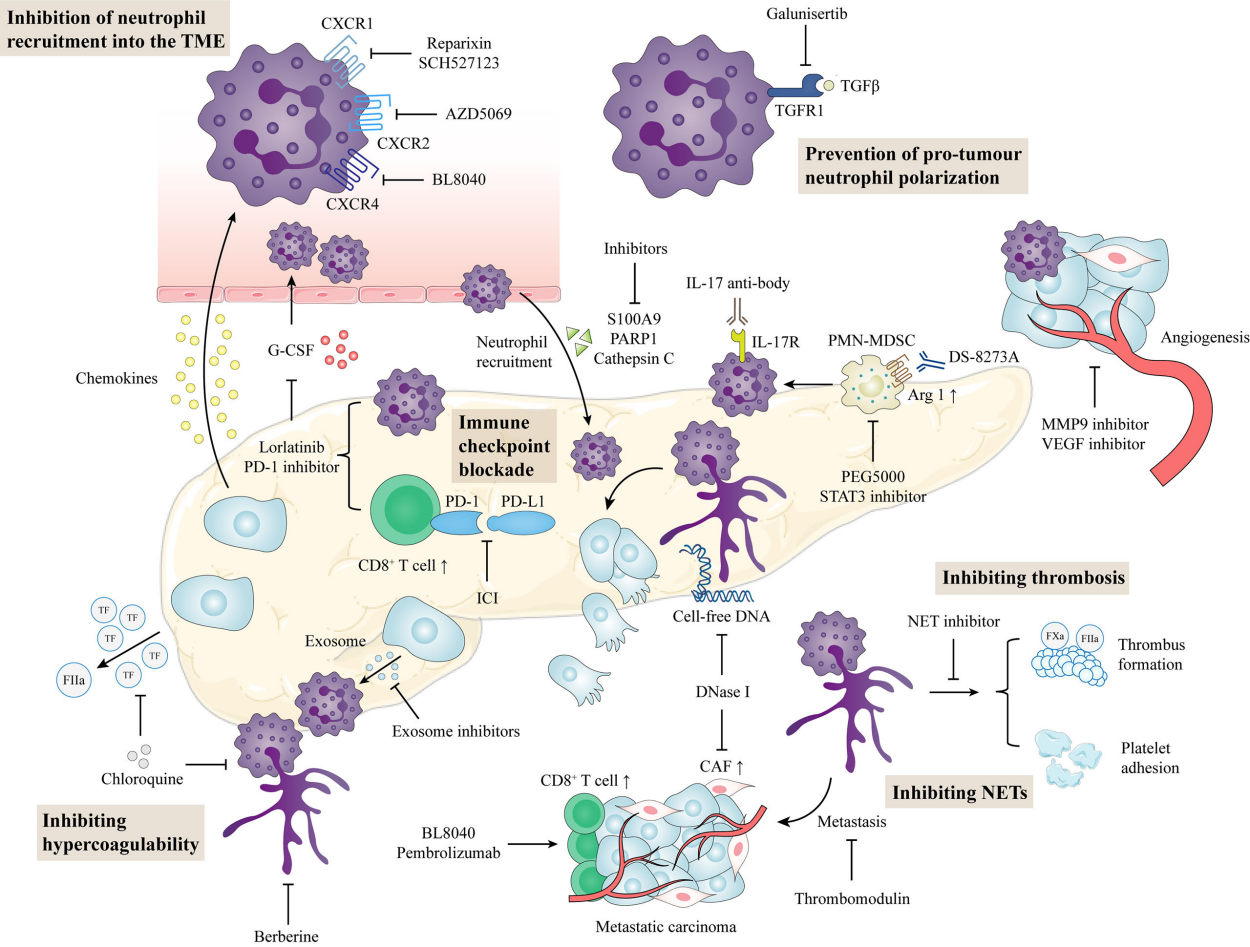
Potential neutrophil-directed therapeutic targets in pancreatic cancer. Inhibition of chemokines and cytokines prevents neutrophil activation and recruitment, thereby reducing neutrophils in the TME. TGF-β inhibitors can reduce the tumor-promoting phenotype of neutrophils. In the TME, targeting neutrophil combined with immune checkpoint blockade can enhance the antitumor function in pancreatic cancer. NET inhibitors prevent cancer cell metastasis, circulating hypercoagulable states, and venous thrombosis formation. TME, tumor microenvironment; CAF, cancer-associated fibroblasts; PD-L1, programmed cell death-ligand 1; PD-1, programmed cell death 1; PMN-MDSC, polymorphonuclear-myeloid derived suppressor cell; NET, neutrophil extracellular trap.

Gemcitabine, which produces anticancer activity by interfering with DNA synthesis in cancer cells, has been the most important chemotherapy drug for patients with pancreatic cancer in the past two decades (78). Due to the poor efficacy of chemotherapeutic drugs in some patients, new clinical treatment strategies are increasingly accepted in the treatment of pancreatic cancer. At present, targeted therapy and immunotherapy is representative of a new generation of cancer therapies, and is also the focus of pancreatic cancer research (79). The exploration of the biological function of immune cells in the tumor microenvironment will lead to more effective therapies to suppress the inflammatory response of the TME using cytokine inhibitors, chemokine inhibitors and immune checkpoint inhibitors to enhance anticancer immunity. TAN targeted therapies have been validated in human cancers (80). The importance of neutrophils in mediating the effects of cancer therapies and the changes in neutrophils during these treatment processes within the TME is an emerging area of research. Several neutrophil-modulating therapies were originally developed for other indications and have effects beyond neutrophils. The current neutrophil modulatory effects of the treatments are summarized in **Figure 3**.

### Chemokine inhibitors

Chemokine systems have been widely considered as potential new drug targets for cancer treatment due to their biological roles in the TME (81). The recruitment and activation of neutrophils is dependent on CXCR2, so CXCR2 is one of the most studied sites of action for neutrophil-targeted therapy. The ligands of CXCR2 mainly include CXCL1, CXCL2, CXCL3, CXCL5, and CXCL8 (82). The receptor-ligand axis of these chemokines can drive the mobilization and recruitment of neutrophils. Therefore, targeting CXCR2 is beneficial for reducing neutrophils in the TME. *In vivo*, blocking CXCR2 inhibits neutrophil mobilization, and the combination of CCR inhibitors and CXCR2 inhibitors enhances the pancreatic cancer response to FOLFIRINOX chemotherapy (83).

Currently, many CXCR4 or CXCR4 ligand inhibitors are in clinical development. For example, the CXCR4 inhibitors LY2510924 and AMD3100 have been evaluated for antitumor activity in combination with other drugs in patients with colorectal and pancreatic cancer (NCT02737072) (84, 85). In a phase II A clinical trial (NCT02826486), investigators evaluated the safety and efficacy of the CXCR4 antagonist BL-8040 in combination with pembrolizumab in metastatic PDAC and found that BL-8040 increased the tumor infiltration of CD8+ T cells. When BL-8040 was combined with pembrolizumab in chemotherapy-resistant patients, the media OS was 3.3 months and the disease control rate was 34.5% in the evaluable population. Additionally, objective response rate, disease control rate, and median response duration were 32%, 77%, and 7.8 months in the cohort that 22 patients received BL-8040 and pembrolizumab with chemotherapy, respectively. These results suggesting that CXCR4 and PD-1 co-inhibition may amplify the benefits of chemotherapy for patients with PDAC (86).

The loss or inhibition of CXCR2 enables the entry of T cells into the pancreatic cancer TME and enhances the antitumor immune function of the TME. In xenograft tumor models, the combination of CXCR2 and PD-1 inhibitors significantly prolonged the survival of mice (87). AZD5069 is a small CXCR2 antagonist that attenuates TGF-β -mediated drug resistance in cancer cells (88). AZD5069 is also evaluated in phase I B and II clinical studies (NCT02583477) for safety and antitumor activity in metastatic PDAC (89). CXCL5, a ligand of CXCR2, induces angiogenesis in pancreatic cancer. Inhibition of CXCL5 with small interfering RNA and neutralizing antibodies reduced tumor growth in a mouse model of pancreatic cancer (63). Although CXCL5 inhibitors have not yet been tested in cancer patients, their blockade of neutrophil recruitment and anti-angiogenesis actions provide a direction for the future treatment of pancreatic cancer patients.

### Cytokine inhibitors

Cytokines are key mediators of cell signaling in the TME (90). Recently, cytokines and cytokine receptors have received extensive attention as targeted therapies for cancer, mainly by inhibiting pro-inflammatory cytokines and pro-tumor cytokines (91). As mentioned above, cytokines in the TME can induce neutrophil differentiation and prolong the lifespan of neutrophils. Therefore, inhibition of these cytokines to prevent neutrophils from differentiating into pro-tumor phenotypes is also one of the current targeted therapies.

TGF-β promoted the differentiation of neutrophils into a pro-tumor phenotype. TGF-β inhibitors mainly target the serine/threonine kinase domain of TGF-β receptor 1; for instance, galunisertib *in vivo* in combination with immune checkpoint inhibitors can significantly inhibit the growth of pancreatic cancer and enhance the antitumor M1 macrophage infiltration in the TME (92, 93). In current phase I B and phase II trials, galunisertib has also shown good tolerability, safety and antitumor activity in unresectable pancreatic cancer (NCT02734160) (94, 95). Selecting predictive biomarkers of TGF-β inhibition in pancreatic cancer patients may be more effective in predicting treatment effect and patient prognosis.

IL-17 can recruit neutrophils and form NETs that reduce cytotoxic CD8+ T cells in the pancreatic cancer TME (74). In mice with IL-17 overexpression, antibodies to IL-17 and IL-17 receptors reduce pancreatic intraepithelial neoplasia and neutrophil infiltration, and antibodies to IL-17 and IL-17 receptors are currently in clinical trials (96, 97).

G-CSF plays an important role in the activation and mobilization of neutrophils. Lorlatinib is a novel, oral tyrosine kinase inhibitor with anticancer activity in ALK- or ROS1-positive cancer patients (98). Lorlatinib prevents G-CSF and GM-CSF from inducing neutrophil migration. In PDAC, lorlatinib specifically targets neutrophils to inhibit cancer cells by regulating the development of neutrophils in bone marrow cells, reducing the accumulation of neutrophils in the TME, and inhibiting tumor tissue fibrosis. When lorlatinib is combined with anti-PD-1, the number of CD8+ T cells increases and CD44+, CD69+, CD8+ T cells are activated, suggesting that lorlatinib improves the response of PDAC to immunotherapy (99). Similarly, a combination of suppressing cytokines that promote neutrophil recruitment and blocking immune checkpoints has been demonstrated in several preclinical trials. For instance, combination of anti-CSF1 receptor, anti-PD-1 and gemcitabine decreased the infiltration of myeloid cells and improved the antitumor effect (100). Some clinical trials that evaluating the safety and activity of cytokine inhibitors combined with immune checkpoint inhibitors are ongoing (NCT02947165, NCT04581343, NCT02777710).

### Inhibition of NETs

NETs are now considered a promising cancer treatment target. Because NETs are involved in angiogenesis, immunosuppression and metastasis of cancer, inhibiting their formation or promoting their elimination has been proposed as a novel therapeutic strategy for cancer (46, 56). There are two main methods to inhibit NET: inhibiting the formation of NET and destroying the structure of NET. NET formation is mediated by peptidyl arginine deiminase 4 (PADI4) and elastase, in which PADI4 promotes the expulsion of chromosomes *via* histone citrullination, but berberine can inhibit PADI4 expression *in vitro* (101, 102). In PADI4-deficient mice, pancreatic cancer growth was shown to be restricted (103). Cancer cells can also release exosomes to stimulate NET formation. Exosomes play a role in intercellular communication by transferring intracellular substances such as proteins, metabolites and nucleic acids to recipient cells (104). Cancer-derived exosomes can transfer factors related to cancer progression and promote tumorigenesis by regulating proliferation, metastasis, immune escape and increasing drug resistance processes (105). Studies have shown that cancer-derived exosomes transfer mutant KRAS to neutrophils, thereby promoting NET formation by upregulating IL-8 (106). Therefore, inhibition of exosome release in the TME is also a potential antitumor strategy.

Suppressing the components of NETs is also one of the strategies for targeting NETs. Serum DNA and citrullinated histone H3 are markers of NET formation. DNase is considered a promising cancer treatment for its ability to degrade circulating free DNA, thereby destroying the structure and function of NET (107). In a preclinical model of pancreatic cancer, the use of DNase I significantly reduced the number of fibroblasts accumulated in liver metastases, thereby attenuating NET-induced cancer invasion and metastasis (108). Thrombomodulin protein can degrade NET-derived high mobility group box 1 through thrombin, thereby inhibiting NET-induced epithelial-mesenchymal transformation and preventing the invasion and metastasis of pancreatic cancer cells (71).

Chloroquine is also a candidate to inhibit NETs. Chloroquine destroys the structure of NETs by inhibiting autophagy of glycosylated end-product receptors in pancreatic cancer (70). A meta-analysis evaluating the clinical value of using chloroquine as an autophagy inhibitor in the treatment of cancers showed that autophagy inhibitor therapy significantly improved the objective response rates, OS and progression-free survival of cancer patients, suggesting that the role of chloroquine in the treatment of pancreatic cancer should also be explored (109).

Additionally, gentamicin inhibits NETs release from human neutrophils and reactive oxygen species inhibitor (diphenyleneiodonium chloride) also reduces NET formation in a concentration-dependent manner (110, 111). These drugs are expected to be further validated the ability to inhibit NETs.

### Treatment of complications and comorbidities for pancreatic cancer

Pancreatic cancer patients often have different diseases or complications in which neutrophils play different roles. Pancreatic cancer patients are in hypercoagulable state, which is directly related to poor prognosis and venous thrombosis (112–114). Citrullinated histone H3 is one of the markers of NETs, and increased expression of citrullinated histone H3 was observed in the thrombi of pancreatic cancer mice. The thrombus weight decreased after using 1A8, an anti-LY6G antibody, to deplete neutrophils and DNase I to deplete NETs (115). Chloroquine inhibits NET formation and reverses NET-mediated platelet activation and aggregation, as well as tissue factor release. Researchers further found that the rate of venous thromboembolism in patients treated with hydroxychloroquine was 9.1%, while that in the control group was 30% (70, 116).

Neutrophil infiltration was increased in pancreatic cancer specimens from patients with type 2 diabetes mellitus (T2DM). Patients with elevated neutrophils had reduced OS (HR=5.44, 95% CI 1.12 to 26.34) (117). Anorexia and muscle breakdown induced by PDAC are also associated with inflammatory stimulation of neutrophils mediated by the CCR2/CCL2 axis (118). Inflammatory processes and immune system contribute to the metabolic diseases (119, 120). Therefore, based on the role of neutrophils in pancreatic cancer and metabolic disease, such as T2DM, inhibition of neutrophils can simultaneously alleviate the progression of both diseases. Combination therapeutic strategy involving multiple immunomodulatory therapies may prove to be more effective.

### Other potential therapeutic targets

Tumor genotypes can affect the TME and play a key role in treatment resistance. In gain-of-function Trp53 mutant mice, intratumoral neutrophil infiltration increased and the numbers of CD3+ T cells, CD4+ T cells and CD8+ T cells decreased. After neutrophil removal, the sensitivity of CD40 agonists combined with chemotherapy and immunotherapy was enhanced (121).

Several bioinformatics analyses have shown a significant correlation between oncogene expression and the infiltration of various immune cells, including neutrophils, suggesting that targeting these genes may also be a future therapy (122).

Chemotherapy induces the invasion of cytotoxic T cells into the liver metastases of pancreatic cancer, but only briefly. Neutrophils lead to tumor cell regeneration in metastases, and reducing neutrophil infiltration or inhibiting the Gas6/AXL signaling axis combined with chemotherapy can inhibit metastatic growth (123).

MDSCs are sensitive to TRAIL receptor 2 agonists. The antitumor efficacy of DS-8273A (an anti- TRAILR2 antibody) was evaluated in a phase I clinical trial (including one pancreatic cancer patient). The results showed that DS-8273A eliminated polymorphonuclear MDSCs (PMN-MDSCs, immature neutrophils) and prolonged progression-free survival (124). STAT3 is involved in the regulation of arginase 1 activity in PMN-MDSCs, leading to immunosuppression in the TME. Inhibition of STAT3 or using human recombinant arginase 1 (PEG5000) may be beneficial to ameliorate this immunosuppressive microenvironment (125, 126).

## Areas of future development

Finding novel therapies is currently a hot spot and future research direction for cancer research. As an indispensable part of tumor development, the TME has become a new therapeutic target. Based on the important role of neutrophils in the TME of pancreatic cancer, it is feasible to target neutrophils in the treatment of pancreatic cancer.

### Limitation

There are still some challenges in the treatment of pancreatic cancer patients based on neutrophil related oncogenic mechanisms. First, specific reductions of neutrophils in peripheral blood and the TME can inhibit tumor growth and metastasis but can expose patients to opportunistic infections. Since cancer patients often suffer from malnutrition, cachexia, and reduced resistance, such treatments are impractical. Next, blocking chemokines and cytokines also affects the recruitment and function of “antitumor phenotype” neutrophils and other leukocytes, resulting in the limited specificity of currently conducted approaches. Thus, only a few studies have focused on the effects of drugs on specific TAN phenotypes. Moreover, most of the drugs that regulate the TME and target TANs have been studied in animal experiments, but clinical evidence in solid tumor patients is insufficient. Although there have been pilot studies showing a substantial anticancer effect of neutrophil-targeting inhibitors, these need to be followed by more clinical trials so that targeting neutrophil-associated sites or specific phenotypes can be a new treatment for patients with pancreatic cancer. There is also a need for studies that investigate the adverse effects of targeting neutrophils in pancreatic cancer.

### Improved future direction

The pancreatic cancer TME is a complex and dynamic structure that directly affects the biological behavior of pancreatic cancer cells at the molecular and clinical levels. Current work focuses on interactions among tumor cells, neutrophils and inflammatory factors. We should study the following aspects in the future.

First, extracellular vesicle-mediated signal transduction between pancreatic cancer cells and immune cells should be intensively studied. Extracellular vesicle can help facilitate an exchange of information within various cells in the TME (127). It has been found that extracellular vesicle-RNA and proteins are involved in the metastasis and chemotherapeutic resistance of pancreatic cancer (128). Inhibition of extracellular vesicle release and uptake in the TME may be another therapeutic option. Second, although immune checkpoint inhibitors have made progress in cancer treatment during the past 10 years, they are only effective in a subset of patients. Since neutrophils can induce the TME to form an immunosuppressive microenvironment, this may be one of the reasons for the poor efficacy of immune checkpoint inhibitors in pancreatic cancer. Therefore, the combination of neutrophil inhibitor therapy and immune checkpoint inhibitor therapy also needs more preclinical studies and clinical trials in the future. Furthermore, cellular metabolism has also emerged as a critical determinant of the function of immune cells in the TME. The metabolism of substances in the TME is not only the result of tumor development, but also the promoting factor of tumor progression (129). A subpopulation of TANs with high glycolytic activity has been found to enhance immunosuppressive and tumor-promoting functions (130). Understanding the metabolic requirements of neutrophils in pancreatic cancer and their effect on the growth, metastasis and immunosuppression of pancreatic cancer will also be a novel research direction upon which to intervene for enhanced immunotherapy.

### Neutrophils in pancreatic inflammation and fibrosis

Since pancreatic cancer are closely related to pancreatic chronic inflammation and fibrosis, it is necessary to explore the role of neutrophils in the process of chronic pancreatic fibrosis. Immune cells, especially myeloid cells, play an important role in the pathogenesis of pancreatitis. GM-CSF-mediated increased neutrophil infiltration is the main reason that STAT5 promotes pancreatic fibrosis and chronic pancreatitis (131). However, CXCR2 inhibitors reversed pancreatic inflammation *in vivo* models (132). It is also one of the future directions to conduct joint research with related chronic diseases in cancer mechanistic studies (89). Therefore, it is important to combine pancreatic cancer and chronic pancreatitis in animal models when doing preclinical studies. Including patients with chronic pancreatitis as a comparison group is also necessary when conducting clinical trials.

## Conclusion

Neutrophils play a role in angiogenesis, metastasis and immunosuppression in pancreatic cancer through interactions with other cells in the TME. Various neutrophil modulation therapies are entering preclinical studies and clinical trials for pancreatic cancer. Precision medicine aims to provide patients with more effective personalized medical services (133). The treatment mode of pancreatic cancer is gradually developing toward targeted therapy and precision medicine. The research and development of neutrophil-based therapeutics and targeting neutrophils in combination with other therapies will benefit more patients with pancreatic cancer. Specifically targeting neutrophil-associated sites will be part of therapies for the next generation of cancer patients.

## Author contributions

WJ, XL, and WZ contributed to the study conception and design. The original draft of the manuscript was written by WJ and XL. WJ and XL contributed to visualization. Review and editing was performed by CX and WZ. WZ provided language help. All authors contributed to manuscript revision, read, and approved the submitted version.

## Funding

This work was supported by the Science and Technology Projects of Chengguan District in Lanzhou, China (2020-2-11-4) and the Traditional Chinese Medicine Scientific Research Project of Gansu Province, China (GZKP-2020-28).

## Acknowledgments

The authors would like to express their gratitude to AJE (www.aje.com) for the expert linguistic services provided.

## Conflict of interest

The authors declare that the research was conducted in the absence of any commercial or financial relationships that could be construed as a potential conflict of interest.

## Publisher’s note

All claims expressed in this article are solely those of the authors and do not necessarily represent those of their affiliated organizations, or those of the publisher, the editors and the reviewers. Any product that may be evaluated in this article, or claim that may be made by its manufacturer, is not guaranteed or endorsed by the publisher.
